# MiR-140-3p inhibits the cell viability and promotes apoptosis of synovial fibroblasts in rheumatoid arthritis through targeting sirtuin 3

**DOI:** 10.1186/s13018-021-02236-5

**Published:** 2021-02-02

**Authors:** Beibei Zu, Lin Liu, Jingya Wang, Meirong Li, Junxia Yang

**Affiliations:** Department of Rheumatology, Xuzhou Central Hospital, No.199, South Jiefang Road, Xuzhou, 221009 Jiangsu Province China

**Keywords:** Rheumatoid arthritis, miR-140-3p, Sirtuin 3, Synovial fibroblasts, Apoptosis

## Abstract

**Background:**

Synovial fibroblasts (SFs) with the abnormal expressions of miRNAs are the key regulator in rheumatoid arthritis (RA). Low-expressed miR-140-3p was found in RA tissues. Therefore, we attempted to investigate the effect of miR-140-3p on SFs of RA.

**Methods:**

RA and normal synovial fibrous tissue were gathered. The targets of miR-140-3p were found by bioinformatics and luciferase analysis. Correlation between the expressions of miR-140-3p with sirtuin 3 (SIRT3) was analyzed by Pearson correlation analysis. After transfection, cell viability and apoptosis were detected by cell counting kit-8 and flow cytometry. The expressions of miR-140-3p, SIRT3, Ki67, Bcl-2, Bax, and cleaved Caspase-3 were detected by RT-qPCR or western blot.

**Results:**

Low expression of miR-140-3p and high expression of SIRT3 were found in RA synovial fibrous tissues. SIRT3 was a target of miR-140-3p. SIRT3 expression was negatively correlated to the expression of miR-140-3p. MiR-140-3p mimic inhibited the MH7A cell viability and the expressions of SIRT3, Ki67, and Bcl-2 and promoted the cell apoptosis and the expressions of Bax and cleaved Caspase-3; miR-140-3p inhibitor showed an opposite effect to miR-140-3p mimic on MH7A cells. SIRT3 overexpression not only promoted the cell viability and inhibited cell apoptosis of MH7A cells but also reversed the effect of miR-140-3p mimic had on MH7A cells.

**Conclusions:**

The results in this study revealed that miR-140-3p could inhibit cell viability and promote apoptosis of SFs in RA through targeting SIRT3.

## Background

Rheumatoid arthritis (RA), a chronic systemic autoimmune disease, is characterized by chronic inflammation of synovium and destruction of joint structure [1, 2]. The morbidity of RA is approximately 5–50 per 100,000 populations, the prevalence of RA rises with the increase of age, and about 0.5–1% of adults in the world suffer from RA [2–4]. The clinical manifestations of RA are symmetrical multiple joint swelling and pain, which can eventually cause joint structure damage, dysfunction, and even disability [5]. In addition, RA not only affects the joints of patients but also affects most organs and systems, including the nervous, pulmonary, cardiovascular, and skeletal systems [6]. Therefore, the chronic physical symptoms and disability adversely affected the physiology and psychological health of the RA patients.

So far, the molecular mechanisms of RA pathogenesis are still largely unknown which causes much uncertainty and difficulty in finding out a credible method for RA treatment. It was proved that the synovial fibroblasts (SFs) of RA play a key role in synovial proliferation, joint inflammation, and cartilage erosion [7]. In RA, SFs are always in a state of chronic activation, and its growth regulation mechanism is blocked resulting in “tumor like growth” of the SFs and its own excessive proliferation [7, 8]. Therefore, to explore a way to induce the apoptosis of excessive-proliferated SFs in RA patients may be a potential approach for the treatment of RA. Increasing evidence has supported that SFs with abnormal miRNA expressions are the critical regulator of the biological function of SFs and in the RA progression [9, 10]. Low-expressed miR-650 was found in SFs of RA patients than that in normal cells, and miR-650 inhibited the proliferation and invasion of SFs by regulating AKT2 [11]. Expression level of miR-613 was downregulated in RA tissues and SFs than that in normal tissues and cells; it could suppress the proliferation and induce apoptosis of SFs by targeting DDK1 [8]. MiR-126 was upregulated in RA patients and promoted the resistance of SFs to apoptosis by targeting PIK3R2 [12]. Study also reported that in tissues of AR patients and mice, the expressions of miR-140-5p and miR-140-3p were significantly downregulated [7]; in addition, miR-140-5p overexpression was proved that it had the ability to inhibit SFs proliferation and induce apoptosis through regulating TLR4 [4, 7]. Currently, the effects and mechanisms of miR-140-3p on the apoptosis of SFs remained to be further investigated.

Therefore, the purpose of this study was to explore the effects and the potential mechanisms of miR-140-3p on apoptosis of SFs in RA.

## Methods

### Tissue samples and ethics statement

Synovial fibrous tissue was gathered from 40 RA patients and 40 normal synovial fibrous, who underwent surgical excision at Xuzhou Central Hospital between March 2018 and March 2019. The study had been reviewed and approved by the Ethics Committee of Xuzhou Central Hospital (Z20180305N), and all patients had signed informed consent and agreed that their tissues would be used for clinical research.

### Cell culture

Human embryonic kidney cell 293T (BNCC353535) and human RASFs (MH7A cell) (BNCC341730) were brought in BeNa Culture Collection (Beijing, China). All cells were cultured in DMEM medium (C11995500BT, Gbico, MA, USA) containing 10% fetal bovine serum (FBS) (10437010, Gbico) at 37 °C with 5% CO_2_ in humid atmosphere. The 293T cells were only used for luciferase reporter assays.

### Transfection

Plasmids overexpressing sirtuin 3 (SIRT3) were ligated into the pcDNA3.1 (60908-1440, Tiandz, Beijing, China). MiR-140-3p mimic (miR113617103112-1-10), inhibitor (miR2151225034459-1-10), mimic control (miR1N0000001-1-5), and inhibitor control (miR2N0000001-1-5) were obtained from RIBOBIO (Guangzhou, China). Before transfection, the MH7A cells were placed into 6-well plates, with each well containing 1.0 × 10^6^ cells and 2 ml medium. After growing overnight until the cell confluence reached 60–70%, 100 μl DMEM medium without FBS was used to dilute 2 μg plasmids, mimic, or inhibitor, while 3 μl lipofectamine 2000 (11668-019, Invitrogen, MA, USA) was added to 100 μl medium, and the two types of medium were co-incubated for 15 min at room temperature. Finally, the mixed liquid was added into cells of each well, and 1.8 ml medium to allow the cells to grow for an additional 48 h.

### Luciferase reporter assays

The fragments of SIRT3-3′-UTR with wide-type (SIRT3-WT) (5′-GTTTCTGTGGCTATGTGTGGTAT-3′) and mutant (SIRT3-MUT) (5′-GTTTCTGTGGCTATGCTGACCAT-3′) binding sites for miR-140-3p were inserted into pmirGLO luciferase Vectors (E1330, Promega, CA, USA). The fragments of SIRT1-3′-UTR with wide-type (SIRT1-WT) (5′-TTTAAATACCTATCACTGTGGTA-3′) and mutant (SIRT1-MUT) (5′-TTTAAATACCTATCAACTCATGA-3′) binding sites for miR-140-3p were also inserted into pmirGLO luciferase Vectors. 293T cell was placed into 48-well plates, with each well containing 3.0 × 10^4^ cells in 300 μl medium. After growing overnight, SIRT3-WT, SIRT3-MUT, SIRT1-WT, or SIRT1-MUT was co-transfected with miR-140-3p mimic or inhibitor into 293T cells using lipofectamine 2000; the cells were then collected and prepared for dual-luciferase reporter assay (Promega). Luciferase activity of cells was determined by GloMax fluorescence reader (Promega).

### Western blot assays

Total protein from the tissues or cells was isolated by RIPA lysis buffer (P0013B, Beyotime, Shanghai, China), and a BCA assay kit (23250, Pierce, MA, USA) was used to detect the concentration of total protein. Finally, total protein (25 μg) was separated in each lane on 10% SDS-PAGE gels (P0052A, Beyotime), electro-blotted and transferred to NC membranes (HTS112M, Millipore, MA, USA). Then, all membranes were incubated with 5% skimmed milk for 1 h at room temperature and further incubated with relative first antibodies: SIRT3 (1:1000, ab223531, 43 kD, Abcam, CA, USA), Ki67 (1:1000, ab92742, 359kD, Abcam), Bcl-2 (1:1000, ab59348, 26kD, Abcam), Bax (1:1000, ab32503, 21kD, Abcam), cleaved Caspase-3 (1:1000, ab2302, 17kD, Abcam), GAPDH (1:1000, ab8245, 36kD, Abcam), and β-actin (1:1000, ab8226, 42kD, Abcam). Next day, HRP-conjugated secondary antibodies (goat anti-mouse IgG secondary antibody (1:5000, ab205719, Abcam) and goat anti-rabbit IgG secondary antibody (1:5000, ab205718, Abcam)) were incubated with the membranes for 1 h at room temperature. Finally, SuperSignal West Pico Chemiluminescent Substrate (34078, Thermo Scientific) was used to incubate the membranes for detecting the signal. The Image Lab™ Software (version 3.0) (Bio-Rad Laboratories Inc., Hercules, CA, USA) was used for the analysis and quantification of the western blot data.

### RNA extraction and RT-qPCR

From clinical samples and MH7A cells, miRNAs were isolated using a miRcute miRNA Isolation Kit (TianGEN, Beijing, China). In brief, for tissue samples, a grinding rod was used to grind the samples in liquid nitrogen in a 1.5-ml centrifugal tube with lysis buffer (provided by kit), while for cells, the cells were collected into 1.5-ml centrifugal tube and added with lysis buffer. Two hundred microliters chloroform (C805334, Macklin, Shanghai) was also added to the cells and shaken for 1 min. After resting the cells for 5 min at room temperature, the cells were centrifuged for 20 min (13,400×*g*) and collected by miRNA solution to a new 1.5-ml tube, into which 75% ethanol (M9082, Macklin) was added and centrifuged for 15 min (13,400×*g*). The sediment was miRNA, and RNase-free H_2_O (ST876, Beyotime) was used to dilute the miRNA sediment. For mRNAs, it was extracted using TRIzol reagent (15596, Invitrogen) following the reference instructions. In brief, the clinical samples and cells were lysed by TRIzol and collected into a new 1.5-ml centrifugal tube (615001, Nest, Wuxi, China); chloroform (C805334, Macklin) was added into a tube and centrifuged for 20 min (14,000×*g*). The supernatant was collected and added with an equal volume of isopropanol (H822173, Macklin) and centrifuged for 5 min (14,000×*g*). RNA sediment was diluted using RNase-free H_2_O.

Then, PrimeScript RT kit (RR037A, Takara, Dalian, China) was used to reverse-transcribe RNA into cDNA according to the reference instructions. Finally, gene expression was determined by RT-qPCR assays using Verso 1-step RT-qPCR Kit (A15300, Thermo Scientific, MA, USA) in ABI 7500 Fast Real-Time PCR System (Applied Biosystems, CA, USA), and the condition of q-PCR was set at 95 °C for 30 s, 60 °C for 30 s, and 45 cycles at 60 °C for 30 s. RNA was quantified by 2^−△△CT^ method. All primer sequences are shown in Table 1.

**Table 1:** RT-qPCR primers

Target gene	Forward primers, 5′-3′	Reverse primers, 5′-3′
miR-140-3p	TGTGTCCTGCCAGTGGTTTT	GTCCGTGGTTCTACCCTGTG
SRIT3	ACCCAGTGGCATTCCAGAC	GGCTTGGGGTTGTGAAAGAAG
Ki67	ACGCCTGGTTACTATCAAAAGG	CAGACCCATTTACTTGTGTTGGA
Bcl-2	GGTGGGGTCATGTGTGTGG	CGGTTCAGGTACTCAGTCATCC
Bax	CCCGAGAGGTCTTTTTCCGAG	CCAGCCCATGATGGTTCTGAT
U6	CTCGCTTCGGCAGCACA	AACGCTTCACGAATTTGCGT
GAPDH	GGAGCGAGATCCCTCCAAAAT	GGCTGTTGTCATACTTCTCATGG

### Cell counting kit-8 (CCK-8) assays

CCK-8 (PA137267, Pierce, MA, USA) was used to test cell viability. After transfection, cells were laid into 96-well plates, with each well containing 1.0 × 10^4^ cells in 100 μl complete medium. After growth for 24 h, 48 h, and 72 h, the cells were incubated by 100 μl CCK-8 reagents (0.5 mg/ml) for 15 min. Finally, the absorbance of each well was detected at 450 nm by an Infinite M200 PRO microplate reader (Tecan Austria GmbH, Austria).

### Flow cytometry

The cell apoptosis was estimated using an Annexin V/PI kit (KGA108, KeyGen Biotech) by flow cytometry according to the reference. In brief, when the transfection and treatment with curcumin were completed, cells were placed into 6-well plates, with each well containing 2.0 × 10^5^ cells with 2 ml of complete medium. After incubation for another 24 h, the cells were collected and incubated with Annexin V for 15 min at a normal atmospheric temperature in dark. Then, cells were incubated with PI for 25 min at a normal atmospheric temperature in dark. Finally, the fluorescence of cells was detected and analyzed by fluorescence-activated cell sorting caliber (FACSCaliburTM; BD Biosciences; San Jose, CA, USA).

### Statistical analysis

Student’s *t* test and one-way ANOVA were applied to analyze the data involved in this study by the SPSS software (version 18.0). LSD and Dunnet’s were used as post hoc tests. Pearson correlation analysis was used to analyze the correlation between the expressions of SIRT3 with miR-140-3p. Mean ± standard deviation indicated the statistical data. All experiments were conducted three times. Statistically, a significant result was labeled by *P* < 0.05.

## Results

### MiR-140-3p was low-expressed in RA synovial fibrous tissues and targeted SIRT3 and SIRT1

**Fig. 1:**
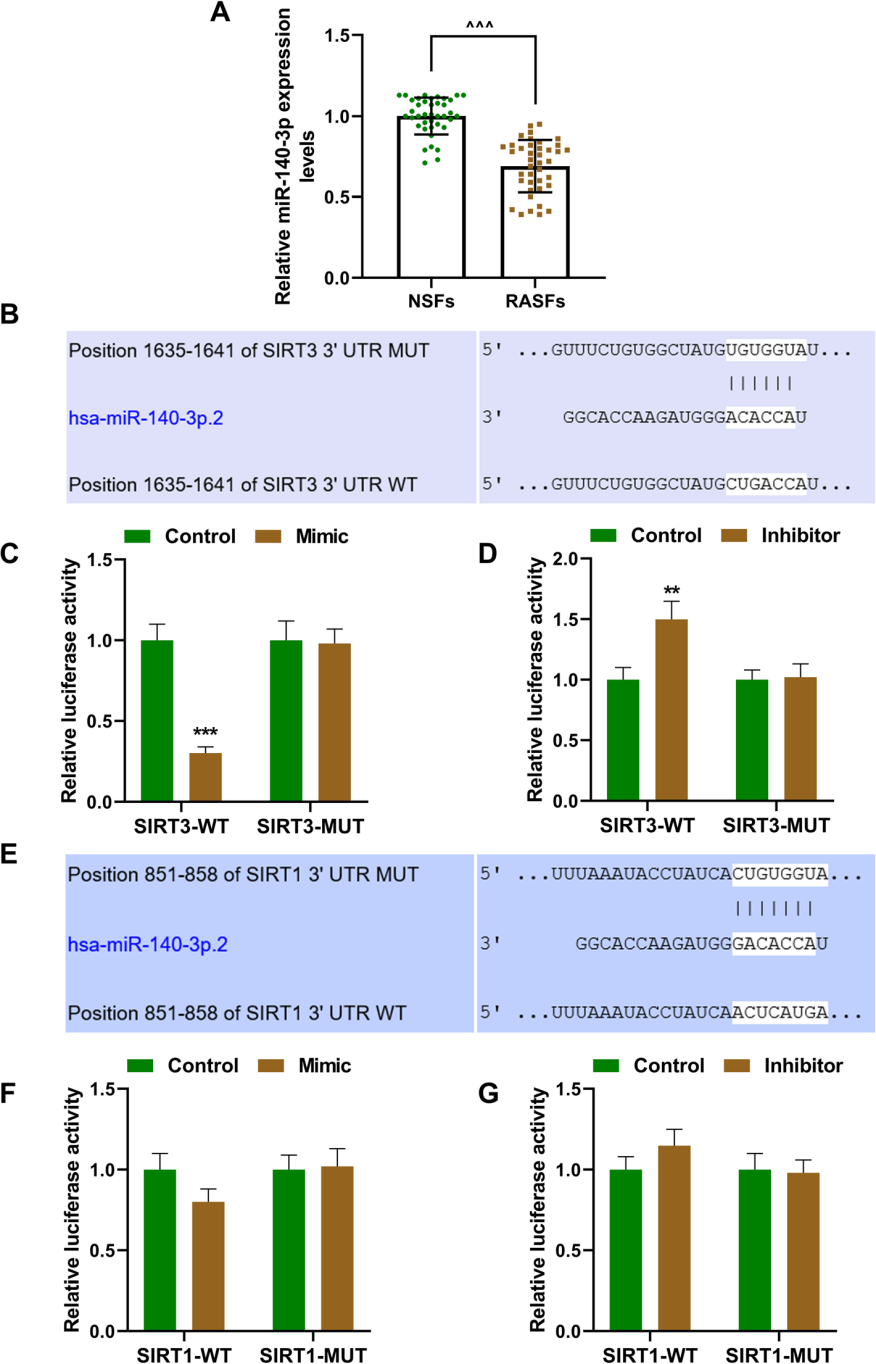
MiR-140-3p was low-expressed in RASFs and targeted SIRT3 and SIRT1. **a** The expression of miR-140-3p in RASFs and NSFs was detected by RT-qPCR; U6 was used as an internal control. (^^^^*P* < 0.01, vs. NSFs). **b**, **e** SIRT3-3′-UTR and SIRT1-3′-UTR containing binding sites of miR-140-3p was predicted by TargetScan. **b**, **c**, **f**, **g** Luciferase assay validated that miR-140-3p targeted SIRT3 and SIRT1 in 293T cells (*P < 0.05, **P < 0.01, ***P < 0.001, vs. control). All experiments were conducted three times. (RASFs, rheumatoid arthritis synovial fibrous tissues; NSFs, normal synovial fibrous tissues; SIRT3, sirtuin 3)

We first detected the expression level of miR-140-3p in RA synovial fibrous tissues (RASFs) and normal synovial fibrous tissues (NSFs), as shown in Fig. 1a, the expression of miR-140-3p was significantly lower in RASFs than that in NSFs (*P* < 0.01). It was also predicted that SIRT3 and SIRT1 were targets of miR-140-3p from the bioinformatics analysis (Fig. 1b, e). Then, we further conducted luciferase reporter assays to verify this prediction. Luciferase activity was decreased after 293T cells were transfected miR-140-3p mimic with SIRT3-WT or SIRT1-WT together as compared to the control groups (*P* < 0.001, *P* < 0.01, respectively), while after being co-transfected miR-140-3p mimic with SIRT3-MUT or SIRT1-MUT there was no difference in the luciferase activities as compared to the mimic control groups (Fig. 1c, f). Luciferase activity was increased after 293T cells were co-transfected miR-140-3p inhibitor with SIRT3-WT or SIRT1-WT as compared to the control groups (*P* < 0.001, *P* < 0.05, respectively), while after being co-transfected miR-140-3p inhibitor with SIRT3-MUT or SIRT1-MUT there was no difference in the luciferase activities as compared to the mimics control groups (Fig. 1d, g). The results of luciferase reporter assays verified that SIRT3 and SIRT1 were targets of miR-140-3p. Considering that the binding ability of miR-140-3p with SIRT3-WT was stronger (Fig. 1c, f), the SIRT3 was chosen for later using in this study.

### SIRT3 was high-expressed in RASFs and was negatively correlated with the expression of miR-140-3p

**Fig. 2:**
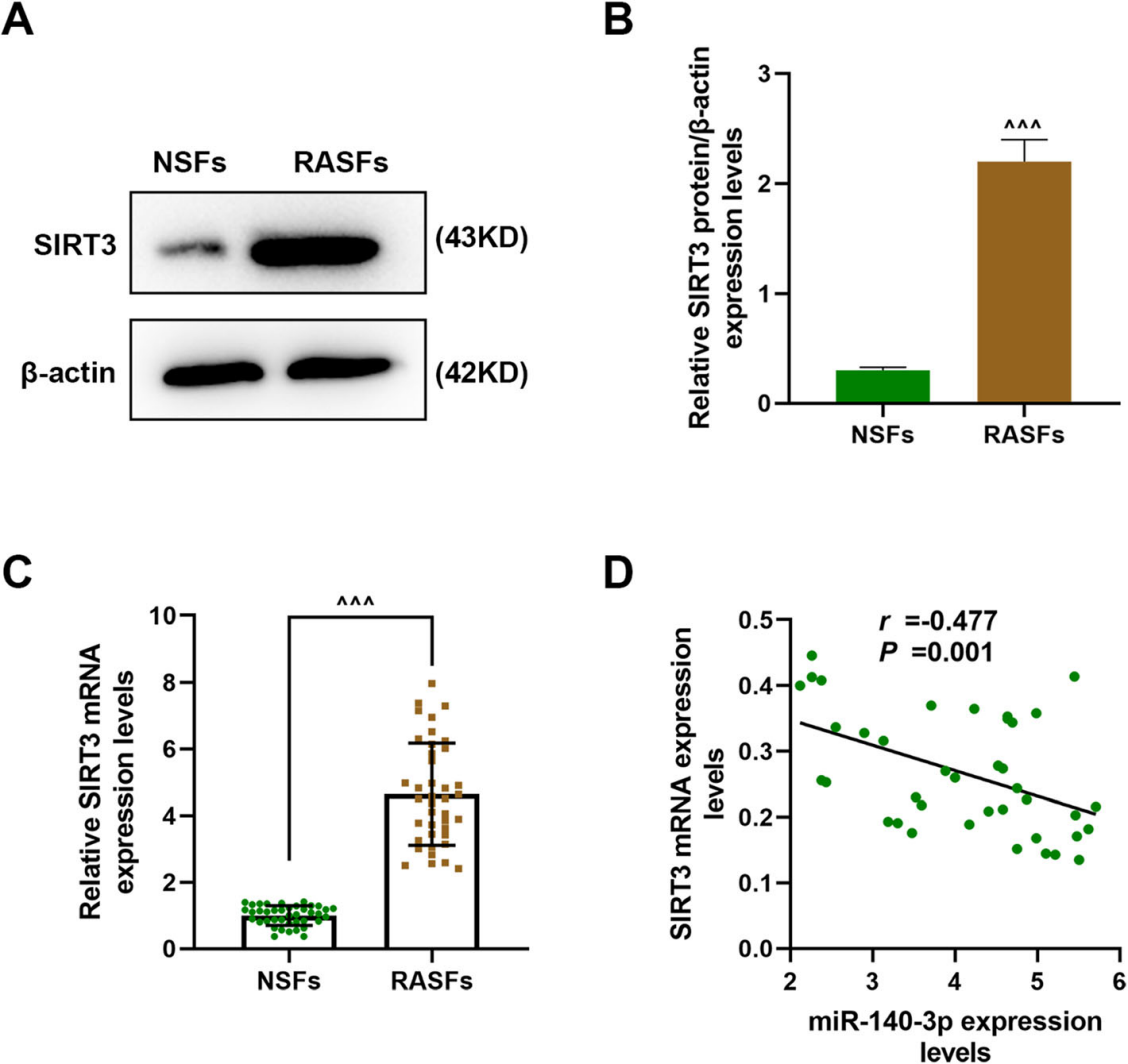
SIRT3 was high-expressed in RASFs and had a negative correlation with the expression of miR-140-3p. **a**, **b** The expression of SIRT3 in RASFs and NSFs was detected by western blot. **c** The expression of SIRT3 in RASFs and NSFs was detected by RT-qPCR. **d** The correlation of the expressions of SIRT3 with miR-140-3p in RASFs was analyzed by Pearson correlation analysis. All experiments were conducted three times. (^^^^^*P* < 0.001, vs. NSFs) (RASFs, rheumatoid arthritis synovial fibrous tissues; NSFs, normal synovial fibrous tissues; SIRT3, sirtuin 3)

We then detected the expression of SIRT3 in RASFs and NSFs (Fig. 2a–c); the protein and gene expressions of SIRT3 were both significantly higher in RASFs compared with NSFs (*P* < 0.001). Mechanically, we analyzed the correlation of the expression of SIRT3 with miR-140-3p in RASFs (Fig. 2d); the results revealed that the expression of miR-140-3p in RASFs was negatively correlated with the expression of miR-140-3p (*r* = −0.477, *P* = 0.001).

### MiR-140-3p regulated the SIRT3 expression, cell viability, and cell apoptosis in MH7A cells

**Fig. 3:**
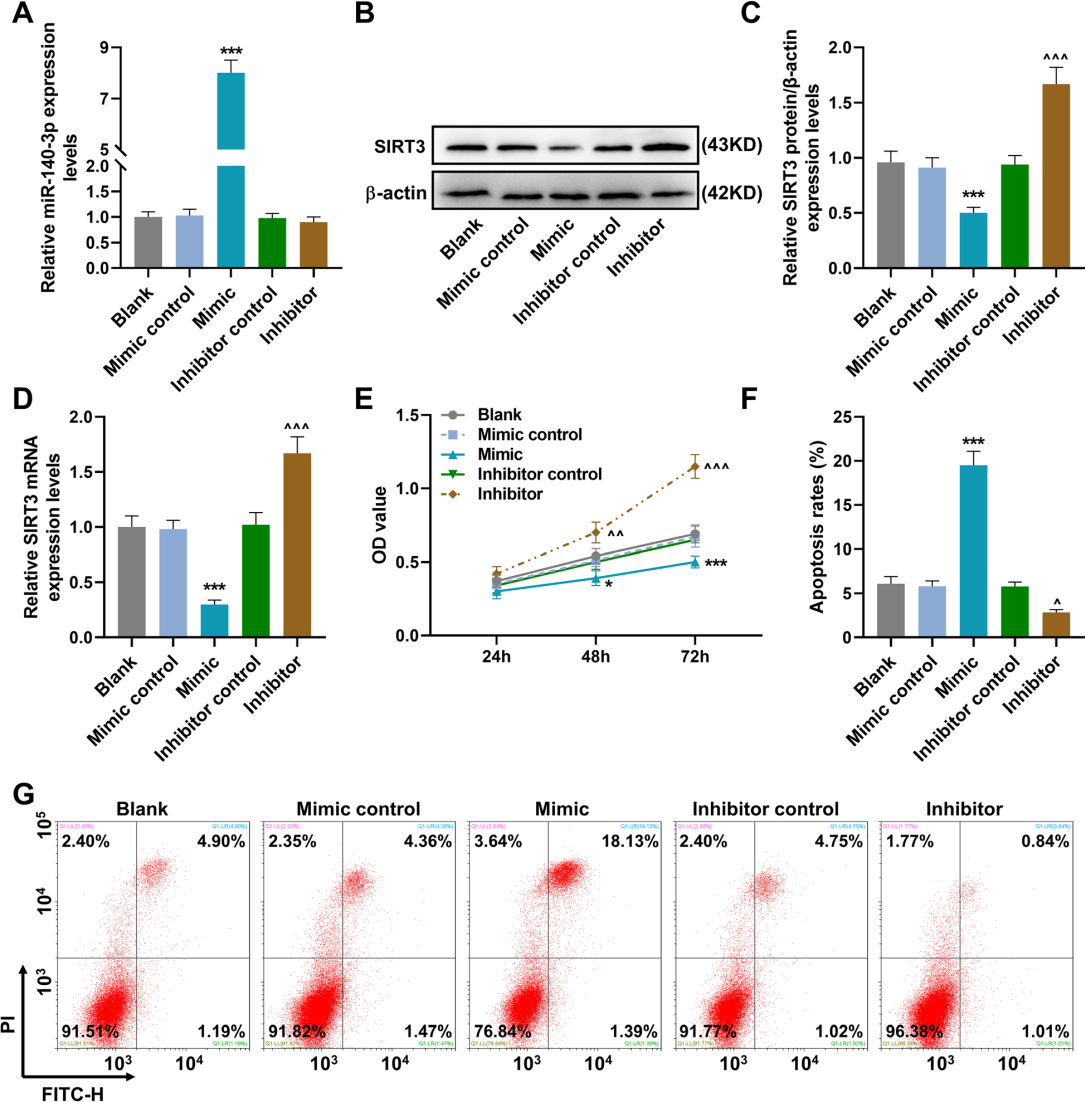
MiR-140-3p regulated the SIRT3 expression, cell viability, and cell apoptosis in MH7A cells. **a** The expression of miR-140-3p in MH7A cells was detected by RT-qPCR; U6 was used as an internal control. **b** The expression of SIRT3 in MH7A cells was detected by RT-qPCR; GAPDH was used as an internal control. **c**, **d** The expression of SIRT3 in MH7A cells was detected by western blot. **e** The cell viability of MH7A cells was detected by CCK-8 assays. **f**, **g** The cell apoptosis of MH7A cells was detected by flow cytometry. All experiments were conducted three times. (*P < 0.05, ***P < 0.001, vs. mimic control; ^^^^*P* < 0.01, ^^^^^*P* < 0.001, vs. inhibitor control) (SIRT3, sirtuin 3)

Here, we observed that after the transfection of miR-140-3p mimic and inhibitor into MH7A cells (Fig. 3a), miR-140-3p mimic remarkably increased miR-140-3p expression as compared to mimic control group (*P* < 0.001), and the inhibitor did not change the expression of miR-140-3p. At the same time, the protein and gene expressions of SIRT3 were both downregulated by miR-140-3p mimic (*P* < 0.001) and upregulated by miR-140-3p inhibitor (*P* < 0.001) as compared to mimic control and inhibitor control (Fig. 3b–d). MiR-140-3p mimic also decreased the viability of MH7A cells after cell culture for 48 h (*P* < 0.05) and 72 h (*P* < 0.001) as compared to mimic control group, while miR-140-3p inhibitor promoted the cell viability of MH7A cells after culture for 48 h (*P* < 0.01) and 72 h (*P* < 0.001) as compared with inhibitor control group (Fig. 3e). We also detected the cell apoptosis changes (Fig. 3f, g); the relative apoptosis rate of MH7A cells was increased by miR-140-3p mimic (*P* < 0.001) and inhibited by miR-140-3p inhibitor (*P* < 0.001) in comparison with mimic control and inhibitor control group.

### MiR-140-3p regulated the expression of apoptosis-related and proliferation-related factors in MH7A cells

**Fig. 4:**
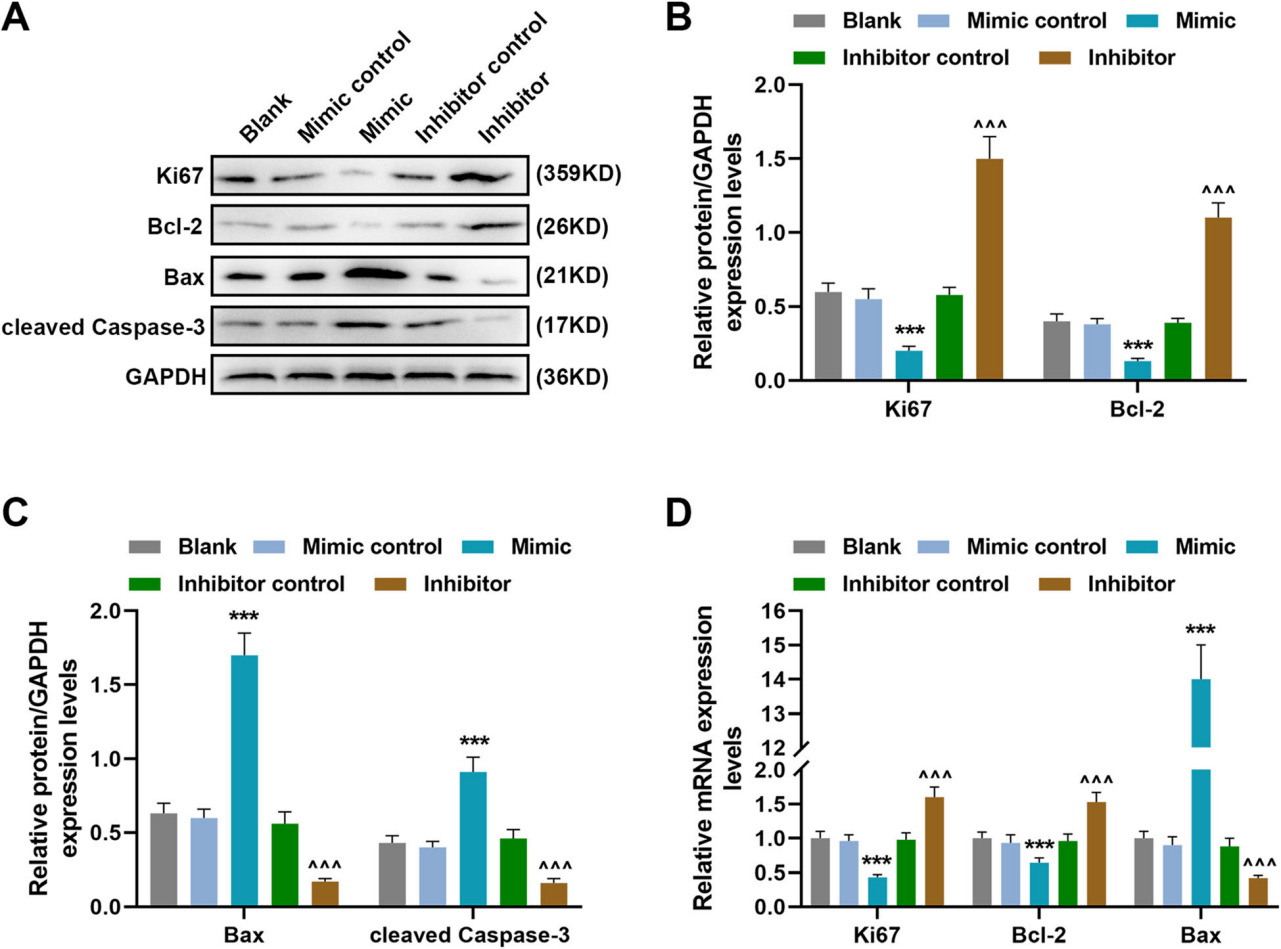
MiR-140-3p regulated the expressions of apoptosis-related and proliferation-related factors in MH7A cells. **a**–**c** The expression of Ki67, Bcl-2, Bax, and cleaved Caspase-3 in MH7A cells was detected by western blot. **d** The expression of Ki67, Bcl-2, Bax, and cleaved Caspase-3 in MH7A cells was detected by RT-qPCR; GAPDH was used as an internal control. All experiments were conducted three times. (***P < 0.001, vs. mimic control; ^^^^^*P* < 0.001, vs. inhibitor control)

Then, we further detected the expressions of proliferation-related and apoptosis-related genes to further confirm current results. MiR-140-3p mimic inhibited the protein expressions of Ki67 and Bcl-2 and increased the expressions of Bax and cleaved Caspase-3 as compared to mimic control group (*P* < 0.001), while miR-140-3p inhibitor increased the protein expressions of Ki-67 and Bcl-2 and decreased the expressions of Bax and cleaved Caspase-3 as compared to inhibitor control group (*P* < 0.001) (Fig. 4a–c). As for the gene expressions of these factors, it had the same tendency with the protein changes (Fig. 4d), which further indicated that miR-140-3p could regulate the proliferation and apoptosis of SFs.

### SIRT3 overexpression reversed the effect of miR-140-3p mimic on SRIT3 expression, cell viability, and cell apoptosis

**Fig. 5:**
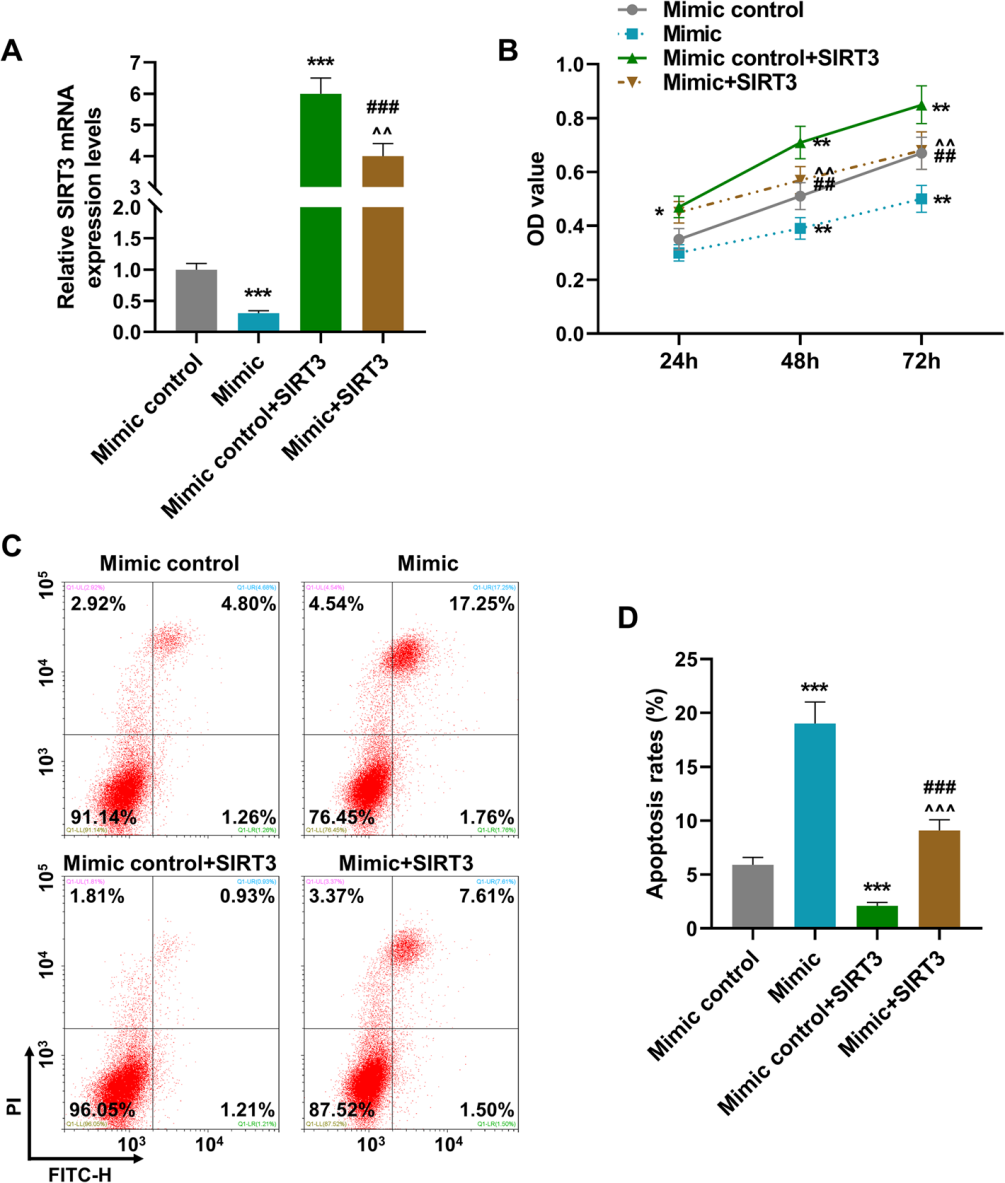
SIRT3 overexpression reversed the effect of miR-140-3p mimic on SRIT3 expression, cell viability, and cell apoptosis. **a** The expression of SIRT3 in MH7A cells was detected by RT-qPCR; GAPDH was used as an internal control. **b** The cell viability of MH7A cells was detected by CCK-8 assays. **c**, **d** The cell apoptosis of MH7A cells was detected by flow cytometry. All experiments were conducted three times. (*P < 0.05, **P < 0.01,***P < 0.001, vs. mimic control; ^##^*P* < 0.01,^###^*P* < 0.001, vs. mimic; ^^^^*P* < 0. 01,^^^^^*P* < 0.001, vs. mimic control +SRIT3). (SIRT3, sirtuin 3)

As exhibited in Fig. 5a, miR-140-3p mimic suppressed SRIT3 expression as compared to mimic control group (*P* < 0.001); after overexpressed SRIT3, the expression of SRIT3 in mimic control+SRIT3 group was obviously upregulated as compared with mimic control group (*P* < 0.001), while after co-transfection with miR-140-3p mimic and SRIT3 overexpression plasmids (mimic+SRIT3) the inhibitory effect of miR-140-3p on the expression of SRIT3 was reversed by SRIT3 overexpression as compared to mimic and mimic control+SRIT3 groups (*P* < 0.001, *P* < 0.01, respectively). As shown in Fig. 5b, miR-140-3p mimic decreased the cell viability of MH7A cells after cell culture for 48 h and 72 h as compared to mimic control group (*P* < 0.01); after overexpressed SRIT3, the cell viabilities in mimic control+SRIT3 group was obviously increased in 24 h, 48 h, and 72 h as compared to mimic control group (*P* < 0.05, *P* < 0.001, *P* < 0.001, respectively), while after co-transfected with miR-140-3p mimic and SRIT3 overexpression plasmids (mimic+SRIT3) the inhibitory effect of miR-140-3p on cell viabilities in 48 h and 72 h was reversed by SRIT3 overexpression as compared to mimic and mimic control+SRIT3 groups (*P* < 0.01). As for the cell apoptosis (Fig. 5c, d), the relative apoptosis rate of MH7A cells was raised by miR-140-3p mimic and SIRT3 overexpression as compared to mimic control group (*P* < 0.001), while after co-transfected miR-140-3p mimic with SIRT3 overexpression plasmids, the promotion effect of miR-140-3p on cell apoptosis was reversed by SIRT3 overexpression as compared with mimic and mimic control+SIRT3 groups (*P* < 0.001).

## Discussion

Synovitis, the basic pathological change of RA, is characterized by the abnormal proliferation of synovial cells which means the imbalance between proliferation and apoptosis of synovial cells [13, 14]. Under normal physiological condition, SFs are located in the lining of synovium, which is involved in normal inflammatory reaction, regulating the function of leukocyte, repairing the joint capsule, and remodeling the matrix in tissue injury [15, 16]. In RA patients, SFs showed abnormal activation, with the characteristics of proliferation and invasion [17]. The abnormal proliferation of SFs in RA patients was the basic of invasion and characterized by the active proliferation and the inhibition of apoptosis [12, 18]. Therefore, SFs is important for the pathogenesis of RA; inhibited proliferation and induced apoptosis of SFs might become an important approach in the treatment of RA.

Although lots of studies have shown that miRNA plays a crucial part in the progress of many kinds of cancer, evidences also showed that the abnormal expression of miRNAs in autoimmune diseases is not only an accidental event [10, 19–21]. Studies from different research teams have shown that miRNAs were widely involved in the regulation of RA pathological and physiological processes, and play an important role in the proliferation, apoptosis, and inflammatory cascade reaction of different SF cells in RA, including miR-613, miR-126, miR-140-5p, miR-451, and so on [4, 8, 12, 22]. Recently, a study reported that the expression of miR-140-3p was low-expressed in the tissues of RA patients and mice [7]. Consistent with the previous research, we also found miR-140-3p was significantly decreased in RA synovial fibrous tissues and SFs. Studies also indicated that overexpression of miR-140-3p had the ability to induce apoptosis of SFs in RA mice. Similarly, in this study, our in vitro results not only exhibited that miR-140-3p mimic inhibited the cell viability and promoted the apoptosis of SFs but only showed that miR-140-3p inhibitor increased the cell viability and suppressed the apoptosis of SFs by regulating the expressions of proliferation- and apoptosis-related factors, which indicated that miR-140-3p indeed possess crucial role in the progression of RA although the mechanisms need further investigation.

Sirtuin (SIRT) family is an evolutionally conserved protein of histone deacetylase III, which consists of seven members (SIRT1 to SIRT7) [23, 24]. Because SIRTs can remove a large number of acyl modifications from cellular proteins, it is now known though to regulate a variety of biological processes: gene expression, cell metabolism, aging, and many others [25]. In addition, the members of SIRT family reported could be targeted by miRNAs to further affect the biological function of series kinds of cells, for example, the upregulated SIRT1 by miR-34a was required for the differentiation of smooth muscle cell [26]; SIRT1 also could be targeted by miR-34a, miR-132, and miR-217 in the progress of astrocytoma [27]; SIRT2 could be targeted by miR-212 to alleviate the ischemic brain injury [28]; SIRT5 is targeted by miR-299-3p to suppress the migration, invasion, and proliferation of liver cancer cells [29]. In addition, SIRT1 is recently reported as a target of miR-140-3p during the apoptosis of SFs [7]. In this study, our bioinformatics and luciferase analysis data proved that SIRT1 and SIRT3 are both the targets of miR-140-3p. And the binding ability of miR-140-3p with SIRT3 was stronger. Upregulated expressions of the SIRT3 in RA patients have been reported which indicated a regulation effect SIRT3 might have in RA [30]. Consistent with the previous study, we also found that SIRT3 was upregulated in RA synovial fibrous tissues and SFs, and the expression of SIRT3 had a negative correlation with the expression of miR-140-3p in RA synovial fibrous tissues. In addition, our results not only exhibited that SIRT3 overexpression increased the cell viability and inhibited the apoptosis of SFs but also showed that SIRT3 overexpression reversed the effect of miR-140-3p on the viability and apoptosis of SFs, which is the first time to reveal the effect of SIRT3 on SFs and the signaling of miR-140-3p effect on SFs.

The limitation of this research was not studying the downstream mechanism of SIRT3, which would be conducted in future.

## Conclusions

In a word, the current study found that miR-140-3p inhibits the cell viability and promotes apoptosis of SFs through targeting SIRT3.

## Data Availability

The analyzed data sets generated during the study are available from the corresponding author on reasonable request.

## Abbreviations

SFs: Synovial fibroblasts
RA: Rheumatoid arthritis
SIRT3: Sirtuin 3
CCK-8: Cell counting kit-8
